# Supplementary material for: Fabrication of Nylon-6 and Nylon-11 Nanoplastics and Evaluation in Mammalian Cells
Source: Nanomaterials (Basel). 2022 Aug 5;12(15):2699. doi: 10.3390/nano12152699 (PMC9370135; doi:10.3390/nano12152699)
Supplement: Supplementary file 1 [file nanomaterials-12-02699-s001.zip › nanomaterials-1838517-supplementary.pdf]

*nanomaterials*

*Supplementary Materials*

# Fabrication of Nylon-6 and Nylon-11 Nanoplastics and Evaluation in Mammalian Cells

**Sai Archana Krovi [1], Maria M. Moreno Caffaro [1], Shyam Aravamudhan [2], Ninell P. Mortensen [1] and Leah M. Johnson [1,*]**

[1] RTI International, 3040 E. Cornwallis Drive, Research Triangle Park, Durham, NC 27709, USA
[2] Joint School of Nanoscience and Nanoengineering, North Carolina A&T State University, 2907 E. Gate City Blvd., Greensboro, NC 27401, USA
* Correspondence: leahjohnson@rti.org

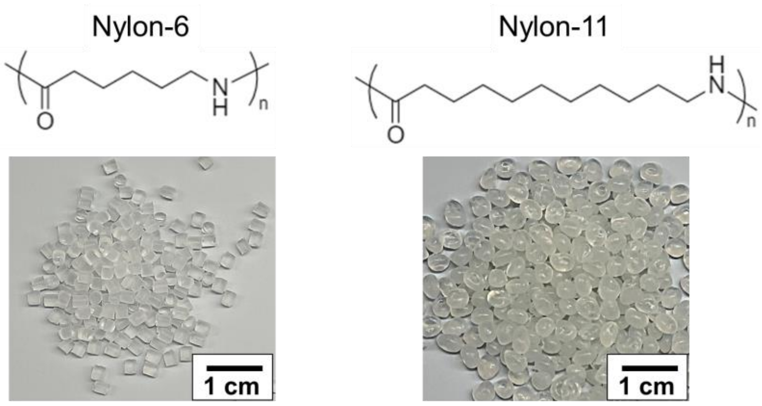


**Figure S1.** Chemical structures of nylon-6 and nylon-11 and their corresponding images of raw pellets used in the fabrication of NPs.

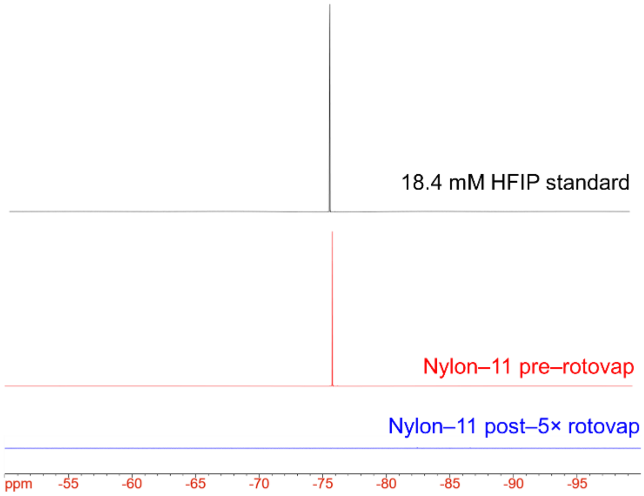


| Sample | HFIP concentration (mM) |
|---|---|
| HFIP standard | 18.4 |
| Pre–rotovap | 122.7 |
| 1× rotovap | 54.5 |
| 2× rotovap | 1.3 |
| 3× rotovap | 0.5 |
| 4× rotovap | 0.2 |
| 5× rotovap | 0.02 |

**Figure S2.** Stacked $^{19}F$ NMR spectra for known concentration of HFIP standard (black), nylon-11 NPs before purification (pre-rotovap, red), and after purification (post-rotovap 5×, blue). A strong HFIP peak was detected in the sample pre-rotovap, but a low peak associated with HFIP was detected in the sample after purification.

**Table S1.** DLS and zeta potential of nylon-11 NP formulations post-fabrication and after each rotary evaporation cycle and re-suspension in ultrapure deionized water.

| NP formulation | Rotovap cycle | Average hydrodynamic diameter ± SD (nm) | Average PDI ± SD | Average zeta potential ± SD (mV) |
|---|---|---|---|---|
| Nylon−11 | Pre−rotovap | 136 ± 45 | 0.16 ± 0.03 | 35.23 ± 1.35 |
| | 1× rotovap | 129 ± 49 | 0.13 ± 0.01 | 35.30 ± 0.94 |
| | 2× rotovap | 131 ± 52 | 0.16 ± 0.02 | 33.73 ± 1.95 |
| | 3× rotovap | 134 ± 56 | 0.16 ± 0.01 | 34.40 ± 0.96 |
| | 4× rotovap | 131 ± 49 | 0.15 ± 0.01 | 33.37 ± 0.62 |
| | 5× rotovap | 127 ± 51 | 0.19 ± 0.00 | 33.07 ± 1.41 |
| Nylon−11 ATRB | Pre-rotovap | 160 ± 49 | 0.08 ± 0.01 | 28.67 ± 0.63 |
| | 1× rotovap | 138 ± 48 | 0.13 ± 0.03 | 33.13 ± 0.81 |
| | 2× rotovap | 141 ± 48 | 0.11 ± 0.04 | 31.33 ± 0.79 |
| | 3× rotovap | 137 ± 35 | 0.05 ± 0.03 | 31.70 ± 1.12 |
| | 4× rotovap | 141 ± 48 | 0.13 ± 0.03 | 33.43 ± 0.65 |
| | 5× rotovap | 142 ± 43 | 0.08 ± 0.01 | 34.30 ± 0.85 |
| Nylon−11 NR | Pre-rotovap | 164 ± 56 | 0.09 ± 0.01 | 30.23 ± 0.49 |
| | 1× rotovap | 142 ± 52 | 0.12 ± 0.03 | 32.67 ± 0.45 |
| | 2× rotovap | 139 ± 45 | 0.12 ± 0.03 | 31.93 ± 0.66 |
| | 3× rotovap | 140 ± 43 | 0.09 ± 0.05 | 31.17 ± 0.45 |
| | 4× rotovap | 145 ± 53 | 0.11 ± 0.01 | 34.50 ± 1.69 |
| | 5× rotovap | 137 ± 39 | 0.08 ± 0.01 | 30.67 ± 0.38 |

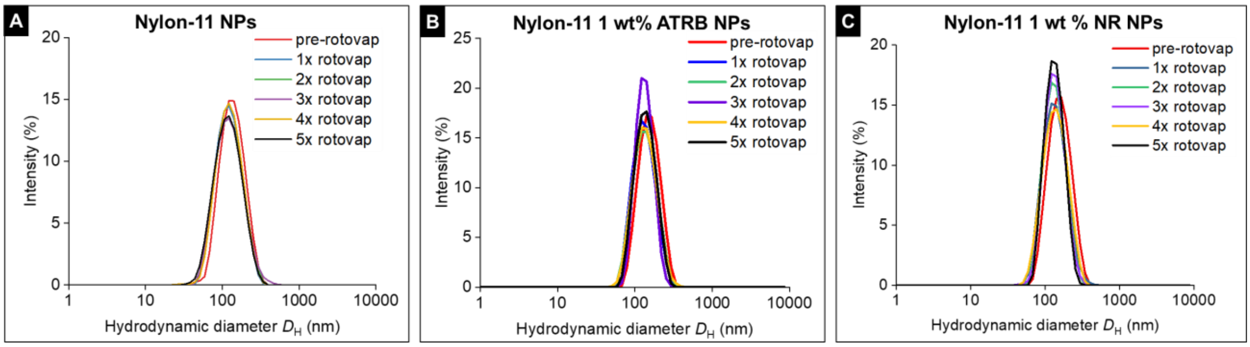


**Figure S3.** DLS measurements of nylon-11 (**A**), nylon-11 ATRB (**B**), and nylon-11 NR (**C**) NPs after each rotary evaporation cycle. Each DLS profile is an average of three measurements.

**Table S2.** Concentration of formic acid in the washed nylon-6 NP formulations determined by formic acid assay.

| NP formulation | Concentration of formic acid (µmol/mL) | Concentration of formic acid (µg/mL) |
|---|---|---|
| Nylon−6 | 1.9 ± 0.5 | 88.2 ± 23.3 |
| Nylon−6 ATRB | 0.72 ± 0.01 | 32.9 ± 0.6 |
| Nylon−6 NR | 0.66 ± 0.06 | 30.2 ± 2.9 |

**Table S3.** DLS and zeta potential of nylon-6 NP formulations post-fabrication and after each wash and re-suspension in 0.5 mg/mL PVA solution in water.

| NP formulation | Sample | Average hydrodynamic diameter ± SD (nm) | Average PDI ± SD | Average Zeta potential ± SD (mV) |
|---|---|---|---|---|
| Nylon−6 | Post−fabrication | 1686 ± 274 | 0.27 ± 0.11 | 5.09 ± 0.62 |
| | After wash 1 | 522 ± 194 | 0.13 ± 0.05 | 12.37 ± 0.58 |
| | After wash 2 | 515 ± 193 | 0.13 ± 0.06 | 26.70 ± 0.51 |
| | After wash 3 (final re−suspension) | 465 ± 132 | 0.10 ± 0.05 | 22.63 ± 0.05 |
| Nylon−6 1 wt% ATRB | Post−fabrication | 755 ± 107 | 0.97 ± 0.04 | 7.39 ± 0.74 |
| | After wash 1 | 567 ± 193 | 0.19 ± 0.04 | 12.27 ± 0.12 |
| | After wash 2 | 508 ± 143 | 0.22 ± 0.01 | 26.30 ± 0.57 |
| | After wash 3 (final re−suspension) | 536 ± 160 | 0.08 ± 0.01 | 21.77 ± 0.26 |
| Nylon−6 0.1 wt% Nile Red | Post−fabrication | 2187 ± 261 | 0.41 ± 0.03 | 3.74 ± 0.38 |
| | After wash 1 | 792 ± 185 | 0.14 ± 0.09 | 9.04 ± 0.40 |
| | After wash 2 | 633 ± 204 | 0.13 ± 0.04 | 18.13 ± 0.37 |
| | After wash 3 (final re−suspension) | 436 ± 139 | 0.17 ± 0.05 | 22.73 ± 0.62 |

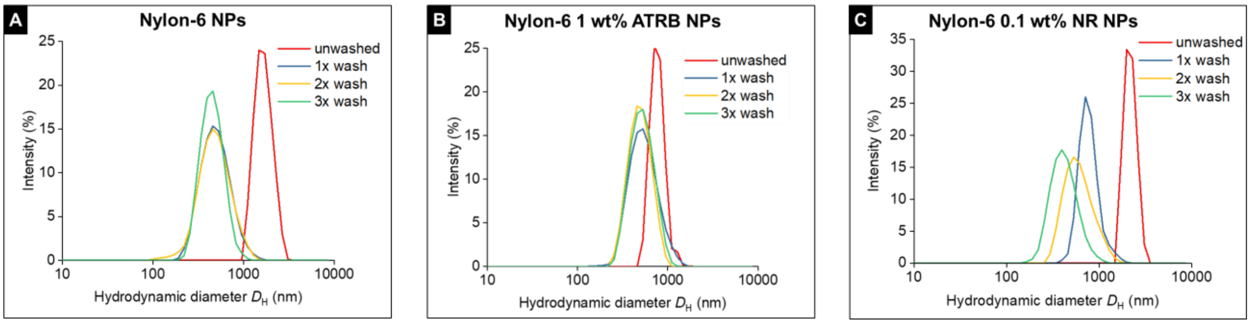


**Figure S4.** DLS measurements of nylon-6 (**A**), nylon-6 ATRB (**B**), and nylon-6 NR (**C**) NPs after each wash and re-suspension in 0.5 mg/mL PVA solution in water. Each DLS profile is an average of three measurements.

**Table S4.** Hydrodynamic diameter, PDI, zeta potential, and fluorophore concentration of fluorophore-loaded nylon-6 NP formulations pre- and post-final wash and re-suspension in 0.5 mg/mL PVA solution in water.

| | | Pre–wash | | | Post–wash | | | |
|---|---|---|---|---|---|---|---|---|
| **Fluorophore** | **Initial fluorophore weight loading** | **Average hydrodynamic diameter ± SD (nm)** | **Average PDI ± SD** | **Average Zeta potential ± SD (mV)** | **Average hydrodynamic diameter ± SD (nm)** | **Average PDI ± SD** | **Average Zeta potential ± SD (mV)** | **Fluorophore concentration (µg/mL)** |
| ATRB | 0.1 wt% | 1723 ± 347 | 0.11 ± 0.14 | 3.25 ± 0.52 | 369 ± 114 | 0.16 ± 0.02 | 25.07 ± 0.12 | 0 |
| | 1 wt% | 755 ± 107 | 0.97 ± 0.04 | 7.39 ± 0.74 | 536 ± 160 | 0.08 ± 0.01 | 21.77 ± 0.26 | 0.7 |
| NR | 0.1 wt% | 2187 ± 261 | 0.41 ± 0.03 | 3.74 ± 0.38 | 436 ± 139 | 0.17 ± 0.05 | 22.73 ± 0.62 | 1.1 |
| | 1 wt% | 1996 ± 470 | 0.26 ± 0.06 | 4.48 ± 0.47 | 351 ± 42 | 0.67 ± 0.29 | 24.17 ± 0.29 | 11.7 |
| Texas Red | 0.01 wt% | 1653 ± 258 | 0.37 ± 0.15 | 5.59 ± 0.62 | 533 ± 148 | 0.22 ± 0.08 | 29.60 ± 0.28 | 0.07 |
| | 0.1 wt% | 1398 ± 219 | 0.39 ± 0.10 | 5.71 ± 0.24 | 548 ± 248 | 0.23 ± 0.01 | 26.03 ± 0.78 | 0.66 |

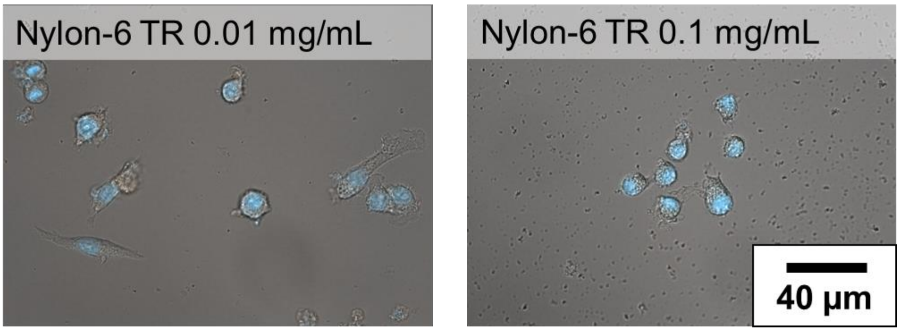


**Figure S5.** Fluorescence microscopy images of RAW 264.7 cells exposed to different concentrations of nylon-6 0.1 wt% TR NPs, exhibiting lack of fluorescence visualization in cells. The blue color results from the DAPI stain for the cellular nucleus.

**Table S5.** The fluorophore concentrations of solutions after removing NPs via centrifugation. The fluorophore concentration was tested immediately after fabrication of NPs (0 days), as well as 7 days and 30 days post-fabrication.

| | Fluorophore concentration (µg/mL) | | |
|---|---|---|---|
| **NP formulation** | **0 days** | **7 days** | **30 days** |
| Nylon−11 ATRB (1 wt%) | 0.008 ± 0.001 | 0.008 ± 0.000 | 0.009 ± 0.001 |
| Nylon−11 NR (1 wt%) | 0.089 ± 0.000 | 0.084 ± 0.004 | 0.087 ± 0.001 |
| Nylon−6 ATRB (1 wt%) | 0.008 ± 0.0003 | 0.008 ± 0.0004 | 0.008 ± 0.0004 |
| Nylon−6 NR (0.1 wt%) | 0.074 ± 0.005 | 0.082 ± 0.004 | 0.087 ± 0.006 |

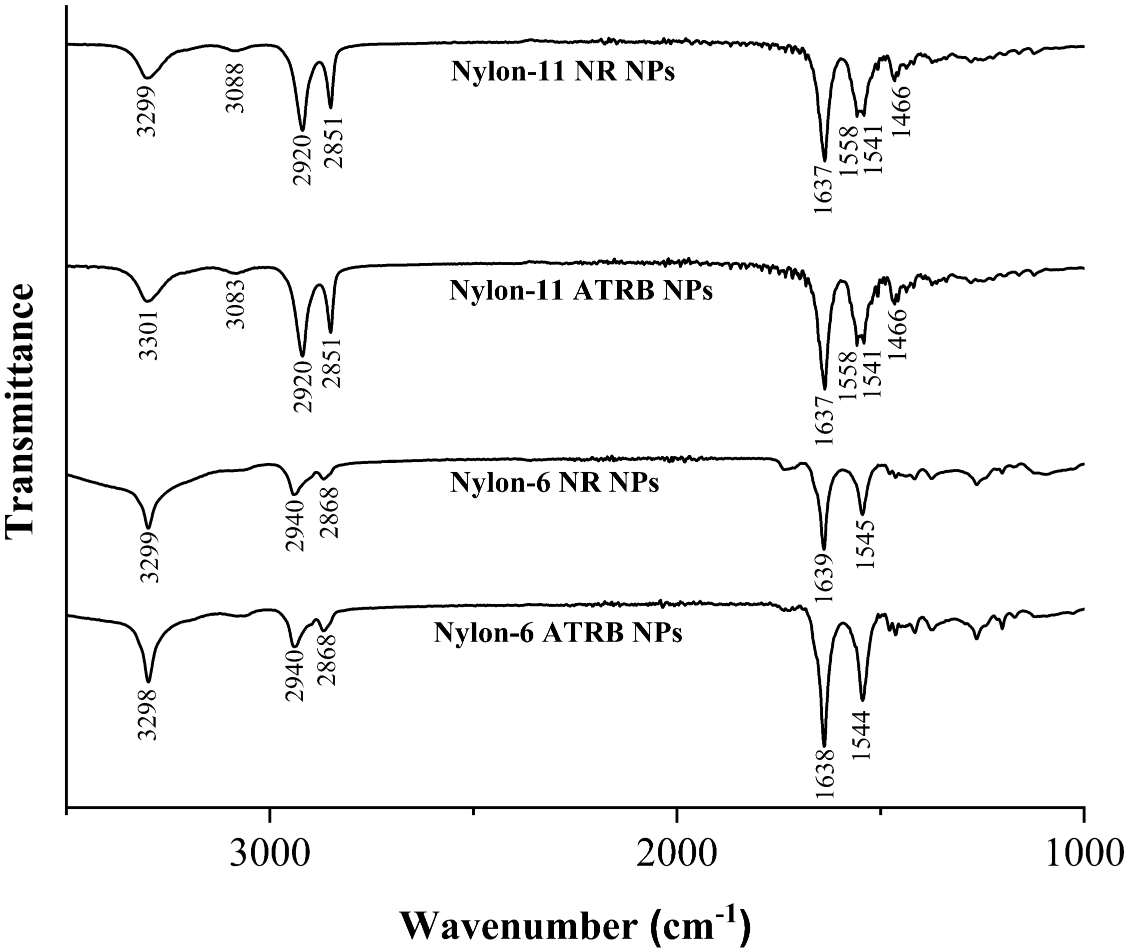


**Figure S6.** FT−IR spectra of fluorescently labeled nylon NPs.

**Table S6.** In vitro Sedimentation, Diffusion and Dosimetry (ISDD) Modeled value for the NPs at the bottom of the well cultured with RAW264.7 cells at 24 h. The concentration and fraction are calculated for the media column from the well bottom to 10 μm above the cell monolayer, reflecting the cell monolayer exposure. "Mean" refers to the mean value across the duration of the study 0 – 24 h, while "24 h" is the value at the 24 h time point.

| NP | Administered Dose (mg/mL) | ISDD modeled values for nanoparticles in the 10 μm immediately above the RAW264.7 cells | | | | | |
|---|---|---|---|---|---|---|---|
| | | Delivered Dose (mg/mL) | | Fraction of administered dose deposited | | Volume of particles per mL | Area of particles per cm$^2$ |
| | | Mean | 24 h | Mean | 24 h | Mean | Mean |
| 50 nm PS | 0.10 | 3.84 | 5.04 | 0.128 | 0.168 | $3.07\times10^{11}$ | $3.07\times10^{8}$ |
| 500 nm PS | 0.10 | 1.19 | 2.19 | 0.0398 | 0.0723 | $6.05\times10^{9}$ | $6.05\times10^{6}$ |
| Nylon-11 NP | 0.10 | 7.50 | 11.0 | 0.250 | 0.367 | $6.91\times10^{9}$ | $6.91\times10^{6}$ |
| Nylon-6 NP | 0.10 | 18.4 | 20.6 | 0.613 | 0.685 | $2.60\times10^{10}$ | $2.60\times10^{7}$ |
